# Multilocus Identification of Indigenous *Trichoderma* Isolates and Their Biocontrol Mechanisms Against *Macrophomina* in Northern Australia

**DOI:** 10.3390/cimb48070654

**Published:** 2026-06-25

**Authors:** Dante L. Adorada, Encarnación E. Adorada, Niroshini Gunasinghe

**Affiliations:** 1 Centre for Crop Health, School of Agriculture, Engineering and Digital Technologies, University of Southern Queensland, Toowoomba, QLD 4350, Australia; encarnacion.adorada@unisq.edu.au (E.E.A.); n.gunasinghe@uq.edu.au (N.G.); 2 School of Agriculture and Food Sustainability, The University of Queensland, Brisbane, QLD 4072, Australia

**Keywords:** DNA barcoding, multigene phylogeny, genomic markers, species delimitation, rhizosphere ecology, saprophytic fungi, sorghum diseases, summer cropping systems

## Abstract

Charcoal rot, caused by the pathogen *Macrophomina*, is becoming an increasing challenge in Australia’s northern cropping systems, with few effective management options available. The use of non-indigenous biocontrol agents raises ecological and regulatory concerns, which highlights the need to identify locally adapted microbial antagonists. In this study, indigenous *Trichoderma* isolates were collected from rhizosphere soils across Queensland and northern New South Wales and characterised using multilocus sequencing (ITS, *tef-1α*, *rpb2*) coupled with phylogenetic analysis. Twenty-six isolates were resolved into six species, dominated by *T. azevedoi* and *T. afroharzianum*. Dual-culture assays revealed substantial variation in antagonistic capacity, with several isolates achieving >70% inhibition of *Macrophomina* growth and maintaining consistent performance across pathogen genotypes. Functional screening indicated that enzyme-associated antibiosis was widespread, whereas volatile-mediated inhibition was restricted to a small subset of isolates. These findings demonstrate that biocontrol potential in indigenous *Trichoderma* populations is highly strain-dependent rather than species-driven. By integrating multilocus identification with functional screening, this study provides a practical framework for selecting locally adapted biocontrol candidates. This work establishes a foundation for developing region-specific biological control strategies and supports a shift toward targeted, strain-level selection for effective management of charcoal rot.

## 1. Introduction

Australia’s northern cropping region, extending from the Central Highlands of Queensland (Qld) to central New South Wales, is characterised by inherently fertile soils, relatively high and variable seasonal rainfall, and a diverse rotation of summer and winter crops [1]. In recent years, several diseases have increased in prevalence across the region, with charcoal emerging as a major constraint to sorghum and other key summer crops. The disease is favoured by prolonged hot and dry conditions, during which *Macrophomina* infects plants through the roots at almost any growth stage, leading to accelerated disease development near maturity and subsequent lodging, often one of the most visible field symptoms. Although formal yield loss quantification *Macrophomina* in Australia remains limited, the pathogen is widely recognised as a significant constraint to sorghum and other summer crops in the northern cropping region [2,3]. Charcoal rot is strongly associated with lodging and reduced grain fill, particularly under conditions of heat and moisture stress, which can lead to substantial production losses. In global systems, *Macrophomina phaseolina* has been reported to cause severe yield reduction in major crops such as soybean, sorghum, and groundnut, with losses occasionally reaching extreme levels under highly conducive conditions [4,5]. Industry-led research initiatives in Australia are currently underway to quantify its economic impact more precisely, reflecting growing concern about its role as an emerging constraint to sustainable grain production.

Management options for charcoal rot are limited due to the pathogen’s endemic nature and wide host range [5]. Consequently, there is increasing interest in biological control as a sustainable management tool. Several studies have demonstrated that *Trichoderma* species as seed treatments can reduce infection by *Macrophomina* [6,7,8].

*Trichoderma* belongs to the family Hypocreaceae [9]. Under the “one fungus–one name” principle, *Trichoderma* is now the accepted name [10]. Species identification requires multilocus phylogenetic analysis due to species complexes [11]. Their biocontrol ability arises from multiple mechanisms, including mycoparasitism, antibiosis, competition, and induction of plant defence responses, which often act in combination depending on environmental conditions and host–microbe interactions [12].

*Trichoderma* species are widely recognised as multifunctional plant-beneficial microorganisms capable of suppressing soilborne pathogens through direct antagonism, competition, and modulation of plant defence responses [12]. These fungi are also known to enhance plant growth and nutrient uptake, making them important components of sustainable agricultural systems.

Globally, *Trichoderma*-based biocontrol systems are widely studied and increasingly adopted as sustainable alternatives to chemical fungicides. These fungi are known to suppress a wide range of soilborne pathogens through mechanisms such as mycoparasitism, antibiosis, competition, and induction of plant defence responses [13]. Plant diseases are estimated to reduce global crop yield by 11–30%, highlighting the need for effective biological control strategies [14]. However, the success of *Trichoderma* as a biocontrol agent is highly strain-dependent and influenced by environmental adaptation, with efficacy varying across agroecological regions. Despite extensive research globally, studies focusing on indigenous *Trichoderma* populations in Australia remain limited. This creates a gap between global advances and local application, reinforcing the importance of identifying and characterising locally adapted isolates. From an ecological perspective, plant-associated microbial communities play a critical role in plant health, nutrient cycling, and disease suppression [15]. This highlights the importance of identifying locally adapted *Trichoderma* isolates that are functionally compatible with the native soil microbiome.

This study aims to identify indigenous *Trichoderma* isolates using multilocus sequencing and to evaluate their biocontrol potential against *Macrophomina* by investigating various mechanisms of action. This includes examining antibiosis through lytic enzymes, the production of inhibitory volatile organic compounds (VOCs), and other functional traits that are important for disease suppression and plant interactions.

## 2. Materials and Methods

### 2.1. Trichoderma Isolation and Identification

**Figure 1:**
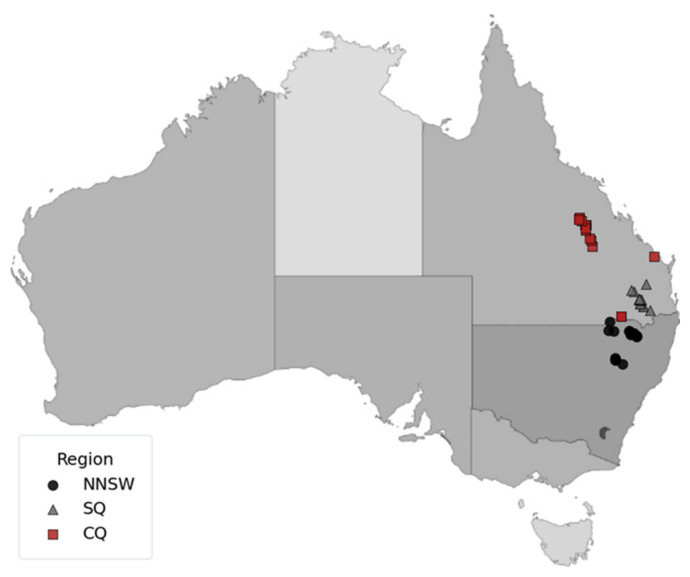
Map of the 55 soil sampling sites in the northern cropping regions (northern New South Wales, southern Queensland and central Queensland) for the *Trichoderma* isolations.

Rhizosphere soil samples were collected from sorghum paddocks in Northern New South Wales (NNSW), Southern Queensland (SQ), and Central Queensland (CQ), for use in the isolation of *Trichoderma* (Figure 1).

A total of 27, 13 and 15 soil samples from different paddocks in NNSW, SQ and CQ, respectively, were collected (Supplementary Table S1). For isolation, a serial dilution for each soil sample was prepared, and the 10^−4^ dilution of each sample was used. Aliquots of each soil suspension were spread, in duplicate, onto Rose Bengal Chloramphenicol Agar (Hi Media, Australia) plates, 1.5% Water Agar (WA) amended with 250 ppm Streptomycin, and half-strength Potato Dextrose Agar (PDA) (Amyl Media Australia) plates amended with 250 ppm Streptomycin [16]. The plates were incubated at 25 ± 1 °C and checked for *Trichoderma* growth between 3 and 7 days. Colonies morphologically consistent with *Trichoderma* that grew on the plates were sub-cultured on PDA until axenic cultures were obtained. Single spore cultures were prepared and kept on PDA plates at 4 °C until use.

The putative *Trichoderma* isolates were cultured in 20 mL potato dextrose broth (PDB) (Difco, Macquarie Park, NSW, Australia) in a 50 mL Falcon tubes for 7 days. The resulting mycelial mats were harvested, blotted dry using sterile absorbent paper towels, and transferred to 1.5 mL microcentrifuge tubes for DNA extraction. Genomic DNA was extracted using the Qiagen™ DNeasy Plant Mini Kit (Qiagen, Clayton VIC, Australia) according to the manufacturer’s instructions.

Molecular identification was based on sequencing of the Internal Transcribed Spacer (ITS) region, RNA polymerase II second-largest subunit (*rpb2*), and translation elongation factor (*tef-1α*) genes. The ITS region was amplified using the universal ITS5 and ITS4 primers [17]. PCR was performed in 25 µL volumes composed of 12.5 µL Hot Start Taq, 8.5 µL nuclease-free water, 0.5 µL each of 10µM forward and reverse primers, and 3 µL genomic DNA (10 ng/µL). Amplification conditions for the ITS region are provided in Supplementary Table S2.

A fragment of the tef-1α gene was amplified using primers ef1 and ef2 [18] under the same reaction conditions, with cycling parameters detailed in Supplementary Table S2. Amplification of the rpb2 gene was performed using primers fRPB2-5 and fRPB2-7cr [19], following the touchdown PCR protocol by Alvarez et al. [20], with conditions provided in Supplementary Table S2.

For each PCR run, a reaction mixture with a known DNA and without a DNA sample was used as a positive and negative check, respectively.

All PCR products were verified by agarose gel electrophoresis prior to sequencing. PCR amplicons were sent to Macrogen (Seoul, Republic of Korea) for sequencing, which used the same primers as those for the PCR amplifications.

### 2.2. Molecular Identification of Trichoderma

Geneious Prime v. 2022.2.2 (Biomatters Ltd., San Diego, CA, USA) was used to trim generated DNA sequence electropherograms manually and to assemble and align sequences. New sequences obtained were deposited in GenBank for the assignment of an accession number. *Trichoderma* identification was done by multi-gene phylogeny, and species identities were confirmed by following the steps described by [18] in their Molecular Identification Protocol for *Trichoderma*. All reference DNA sequences, including ex-type isolates to use in phylogenetic studies, were retrieved from the library of NCBI (http://www.ncbi.nlm.nih.gov, Accessed on 15 June 2025).

A multigene phylogenetic analysis was performed, including ex-type isolates of known *Trichoderma* species and sequences generated in this study to identify isolates to the species level. Sequences of ex-type isolates or reliable reference isolates of *Trichoderma* (when sequences for ex-type were not available) were retrieved from NCBI’s GenBank nucleotide database (Table 1). All reference sequences and new sequences generated in this study were aligned using MAFFT v7.450 within Geneious Prime for three loci separately. Sequences were then filtered and trimmed, avoiding missing data at either end of alignments. The phylogenetic relationships of the representative reference species from different *Trichoderma* species with isolates from this study were reconstructed using a concatenated three-locus dataset (*tef-1α*, *rpb2*, ITS). The dendrogram was constructed using a concatenated data matrix of 20,103 bp, which included partial gene sequences of *tef-1α* ( 630 bp), *rbp2* (863 bp) and ITS (610), and reference sequences from 50 *Trichoderma species*, with *Nectria eustromatica* CBS 125578 as the outgroup taxon. The tree was generated by treating each gene region as a separate partition using Maximum Likelihood (ML) and Bayesian Inference (BI) analyses. Maximum Likelihood (ML) trees were obtained with RAxML v8 [21] available as a plugin in Geneious Prime. Analysis was implemented with the GTR-GAMMA evolution model with 1000 bootstrap replicates. Bayesian Inferences for the concatenated data matrices were performed using MrBayes 3.2.6 [22] in Geneious after selecting the best-fit nucleotide models using the Akaike information criterion in MrModeltest v 2.3. (HKY+I+G for tef-1α, SYM+I+G for rbp2 and GTR+I+G for ITS1). Posterior probabilities were calculated using Markov Chain Monte Carlo (MCMC).

### 2.3. Laboratory In Vitro Bioassay for Antagonism

The three strains of *Macrophomina* used in the assay were BRIP70719 (NNSW), BRIP70725 (SQ), and BRIP70713 (CQ), obtained from the Queensland Plant Pathology Herbarium and selected for their aggressiveness. The cultures were maintained on PDA, incubated at 25 °C for 7 days before use in the study.

The molecularly identified *Trichoderma* isolates, robustly resolved into six *Trichoderma species*, were screened for their ability to inhibit mycelial growth of *Macrophomina* by in vitro dual culture bioassays. A 6 mm diameter mycelial disc from the peripheral region of the growing colonies of a one-week-old culture of *Trichoderma* and *Macrophomina* was placed on opposite sides of the plate at approximately 10 mm from the edge of the 9 cm diameter Petri plates. In control plates, without *Trichoderma*, a sterile agar disc was placed on opposite sides of the *Macrophomina* inoculated disc. The plates were incubated at 25 °C and observed after 7 days. The radial fungal growth (in mm) of the pathogen towards the opposite colony was measured, and the index of antagonism as per cent growth inhibition of *Macrophomina* was calculated using the formula: PI = (C − T)/C × 100, where C = Growth of test pathogen in absence of antagonist (mm), and T = Growth of test pathogen in presence of antagonist (mm) [23]. A two-factor Completely Randomised Design (CRD) was used with four replications, with Factor 1 comprising the three *Macrophomina* strains and Factor 2 comprising the 26 *Trichoderma* isolates.

### 2.4. Biocontrol Mechanisms of Indigenous Trichoderma

A total of 26 indigenous *Trichoderma* isolates identified in the previous experiment were evaluated for their biocontrol mechanisms. Protease, cellulase, and chitinase activities were assessed using plate-based assays as a semi-quantitative screening approach, in which enzyme activity was inferred from the presence and relative intensity of halo zones or colour changes in the respective media. Phosphate solubilisation and ligninolytic enzyme activity were similarly evaluated using standard qualitative plate assays. Detailed media compositions and assay conditions are provided in Supplementary Table S3.

The production of protease was determined based on the Skim Milk Agar Assay by Li et al. [24]. Screening of cellulase producers was done with the Carboxymethyl Cellulose CMC agar as described in previous reports [25,26,27,28]. Chitinase detection in the *Trichoderma* isolates was performed as the Chitin Agar Assay described by Agrawal and Kotasthane [29]. Screening for the relative efficiency of *Trichoderma* isolates to solubilise phosphate was carried out on plates with Pikovskaya’s medium [30,31,32] or NBRIP medium [33] based on the production of organic acids into the surrounding medium.

The qualitative method, Bromophenol Blue Plate Agar Assay, as described by Kameshwar and Qin [34], was followed in identifying *Trichoderma* species that secrete lignin-modifying laccase enzyme [35]. The Azure B Agar Assay was used in screening the *Trichoderma* isolates for lignocellulose-degrading enzyme production [27,28]. The Volatile organic compounds (VOCs) were assessed using an inverted plate assay, where *Trichoderma* and *Macrophomina* cultures were grown in paired plates without physical contact [36,37,38]. Growth inhibition of the pathogen was measured after 7 days and compared with controls. Detailed experimental conditions are provided in Supplementary Table S3.

All the assays described above were performed in four replications, except for the last one, which was conducted with five replications. Each assay was repeated once under identical conditions. These assays were intended as preliminary functional screening tools and did not include quantitative measurements (e.g., halo diameter), limiting the ability to directly compare enzyme production among isolates.

Detailed methodological descriptions have been moved to Supplementary Materials to improve manuscript clarity while maintaining reproducibility.

### 2.5. Statistical Analysis

All data were analysed using analysis of variance (ANOVA) appropriate for a completely randomised design (CRD). Significance was determined at the 5% probability level unless otherwise stated. Prior to analysis, data were assessed for normality using the Kolmogorov–Smirnov test and inspected for homogeneity of variance to ensure that assumptions of ANOVA were met. Where necessary, data transformations were applied.

For dual-culture assays, a two-factor ANOVA was conducted, with *Trichoderma* isolate and *Macrophomina* strains as fixed factors. For other assays, one-way ANOVA was used as appropriate. Mean comparisons were performed using Tukey’s Honestly Significant Difference (HSD) test at *p* ≤ 0.05 to separate treatment effects.

All statistical analyses were performed using Genstat (24th Edition VSN International), following verification that model assumptions were met.

Generative artificial intelligence (GenAI) tools were used to assist with figure preparation and language refinement. All statistical analyses were conducted independently using standard software.

## 3. Results

### 3.1. Identified Trichoderma Species

**Figure 2:**
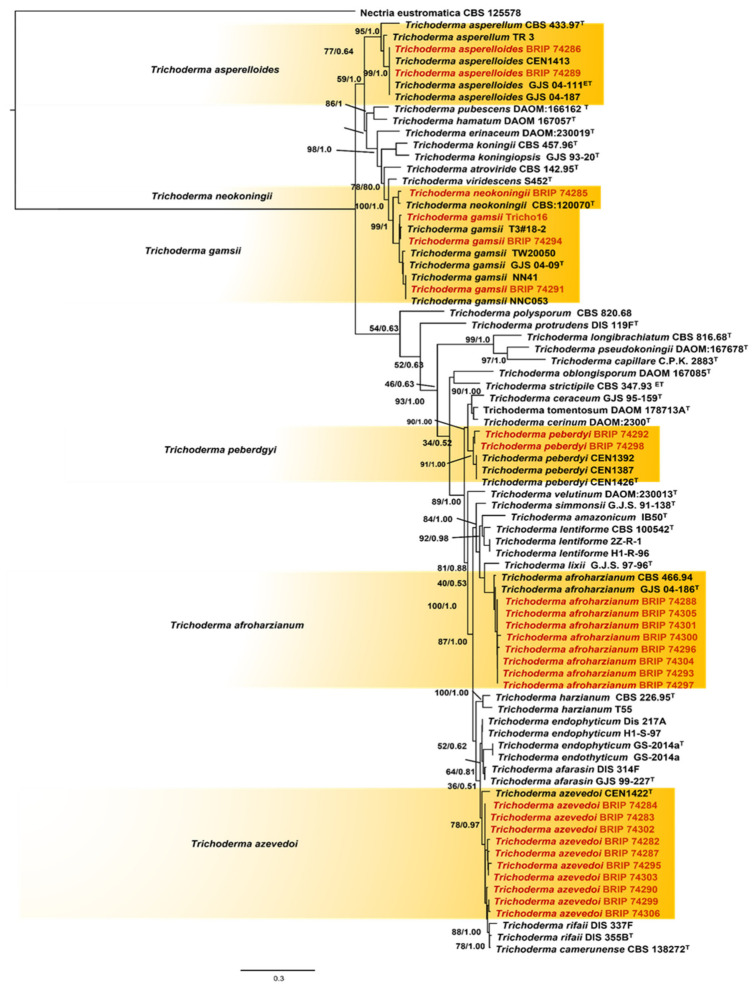
Phylogenetic tree generated by the maximum likelihood analysis using the concatenated sequences of ITS1, *rpb2* and *tef1* gene loci of the genus *Trichoderma.* Maximum Likelihood Bootstrap values ≥ 70% (left) and Bayesian posterior probability values ≥ 0.9 (right) are indicated at nodes (MLBP/BIBP). [DA6.1] *Nectria eustromatica* CBS 125578 was chosen as the outgroup. Newly sequenced and identified *Trichoderma* isolates are indicated by red-coloured fonts.

A total of 26 *Trichoderma* isolates were recovered from rhizosphere soil collected across northern NSW (NNSW), southern Queensland (SQ), and central Queensland (CQ). Multilocus sequencing of the internal transcribed spacer (ITS) region, translation elongation factor-1α (*tef-1α*), and RNA polymerase II second-largest subunit (*rpb2*) gene loci (Table 1), combined with phylogenetic analysis incorporating type culture sequences (Figure 2), enabled taxonomic identification of all isolates. Based on this analysis, the 26 isolates were robustly resolved into six *Trichoderma* species: *T. azevedoi*, *T. afroharzianum*, *T. gamsii*, *T. asperelloides*, *T. peberdyi*, and *T. neokoningii*. Specifically, ten isolates were classified as *T. azevedoi*, eight as *T. afroharzianum*, three as *T. gamsii*, two each as *T. asperelloides* and *T. peberdyi*, and one as *T. neokoningii*. Multilocus phylogenetic analysis provided high-resolution species discrimination and confirmed the dominance of *T. azevedoi* and *T. afroharzianum*, which together comprised most isolates.

This distribution suggests that these species are widely adapted to agroecosystems across the northern cropping region of Australia, whereas the other taxa were less frequently recovered and may represent more specialised or locally distributed populations.

### 3.2. Laboratory In Vitro Bioassay of Trichoderma Antagonism

**Figure 3:**
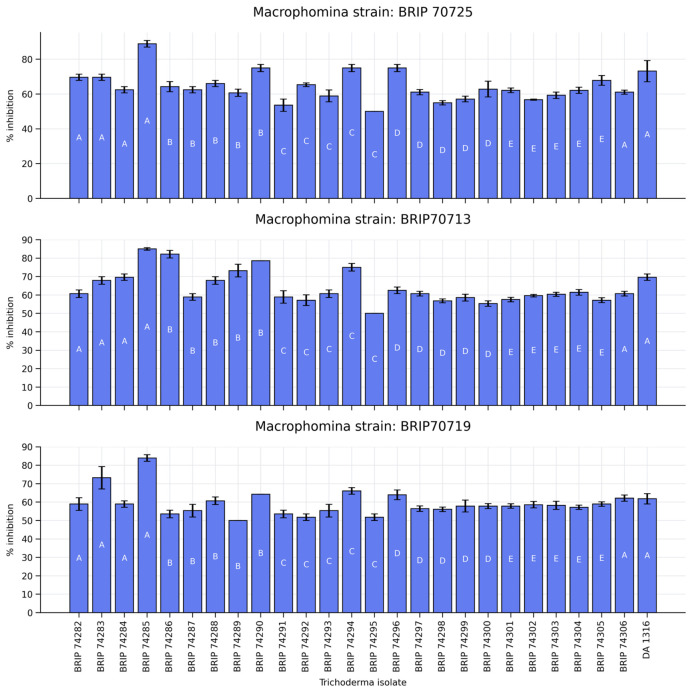
Mycelial growth inhibition (%) of *Macrophomina* strains BRIP 70725 (SQ), BRIP 70713 (CQ), and BRIP 70719 (NNSW) by *Trichoderma* isolates in dual-culture assays. Species identities and BRIP assignments of *Trichoderma* isolates are provided in Table 1. Bars represent mean percentage inhibition across replicates, and error bars indicate ± standard error (SE). All panels share a common scale to allow direct comparison among isolates. Different letters indicate significant differences among *Trichoderma* isolates within each *Macrophomina* strain according to Tukey’s HSD test (*p* ≤ 0.05), with letters assigned independently for each panel.

Dual-culture assays revealed a highly significant effect of *Trichoderma* isolates on mycelial growth inhibition of *Macrophomina* (*p* < 0.001), with a strong and highly significant interaction between *Trichoderma* isolates and *Macrophomina* strains (*p* < 0.001; two-way ANOVA) based on percentage growth inhibition (Figure 3). Significant differences among *Trichoderma* isolates were identified using Tukey’s HSD test (*p* < 0.05), allowing classification of isolates into statistically distinct performance groups. These findings indicate substantial variability in antagonistic activity among *Trichoderma* isolates, as well as differential responses depending on pathogen strain.

When inhibition values were pooled across *Macrophomina* strains, several *Trichoderma* isolates consistently displayed high antagonistic activity. In particular, BRIP74285 (*T. neokoningii*), BRIP74290 (*T. azevedoi*), BRIP74294 (*T. gamsii*), and BRIP74283 (*T. azevedoi*) exhibited the greatest overall suppression of mycelial growth, each achieving more than 70% inhibition. These isolates ranked as the most effective antagonists and demonstrated consistently high levels of inhibition across experiments. Across individual *Macrophomina* strains, *Trichoderma* isolate performance remained generally consistent in rank, although the magnitude of inhibition varied. Notably, BRIP74285 and BRIP74294 showed consistently high inhibitory activity across multiple pathogen isolates, suggesting potential for broad-spectrum activity. This stability across pathogen genotypes suggests that these isolates possess robust and reliable mechanisms of pathogen suppression.

In contrast, several isolates exhibited moderate or strain-dependent inhibition profiles, reflecting significant isolate × pathogen interactions. These differential responses suggest that the efficacy of certain *Trichoderma* isolates may depend on specific host–pathogen combinations, highlighting the importance of accounting for pathogen variability in biocontrol screening. Ranking isolates based on overall antagonistic strength enabled direct comparison across *Macrophomina* strains and facilitated identification of candidate isolates with stable and repeatable inhibitory activity. Importantly, the observed antagonistic capacity was not restricted to isolates from specific geographic locations, indicating that effective isolates are broadly distributed and may have potential for application across multiple agroecological zones.

### 3.3. Biocontrol Mechanisms of Trichoderma Species

**Figure 4:**
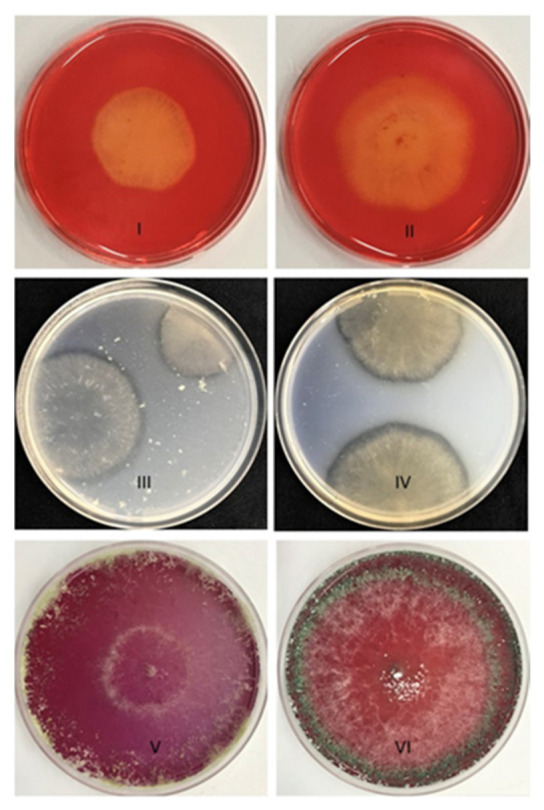
Cellulolytic zone on CMC Agar flooded with Congo red and NaCl in (**I**) BRIP74291_*T. gamsii* and (**II**) BRIP74286 _*T. asperelloides*. Clear halo regions on 1.5%Skim Milk plates of (**III**) BRIP74285_*T. neokoningii*, and (**IV**) DA1316_*T. gamsii*. *Trichoderma* isolates (**V**) DA1316_*T. gamsii* and (**VI**) TBRIP74302_*T. azevedoi* with chitinase activity on medium supplemented with colloidal chitin.

The *Trichoderma* isolates tested showed several traits associated with their potential biocontrol activity (Table 2). Eleven isolates utilised cellulose as a sole carbon source, indicated by the yellow opaque areas of CMC degradation. Chitinases were likely produced by ten *Trichoderma* isolates as indicated by colour changes of the yellow media to purple, which resulted in the breakdown of chitin into N-acetyl glucosamine, causing a shift in pH towards alkalinity, leading to a change of colour of pH indicator dye bromocresol purple (BCP) from yellow to purple zone in the region of chitin utilisation (Figure 4). Nearly all the *Trichoderma* isolates except *T. gamsii* (BRIP 74290) and *T. gamsii* (BRIP 74294) have transparent zones, indicating the ability to produce proteases on the skim milk agar.

In contrast, no transparent halo on fungal colonies was observed on Pikovskaya’s and NBRIP medium, indicating that none of the *Trichoderma* isolates tested could utilise Ca_3_(PO_4_)_2_ as a source of insoluble phosphorus. No ligninolytic enzyme activity was detected in any of the isolates under the conditions tested. None of the *Trichoderma* isolates exhibited decolourisation of Azure B or Bromophenol Blue media, indicating an absence of detectable laccase, lignin peroxidase, or manganese peroxidase activity. Although all isolates were able to grow on these media, no clear or decolourised halo zones associated with ligninolytic enzyme production were observed. These results suggest that direct antibiosis mediated by lytic enzymes (e.g., protease, cellulase, and chitinase) is the dominant mechanism of antagonism under the conditions tested.

**Figure 5:**
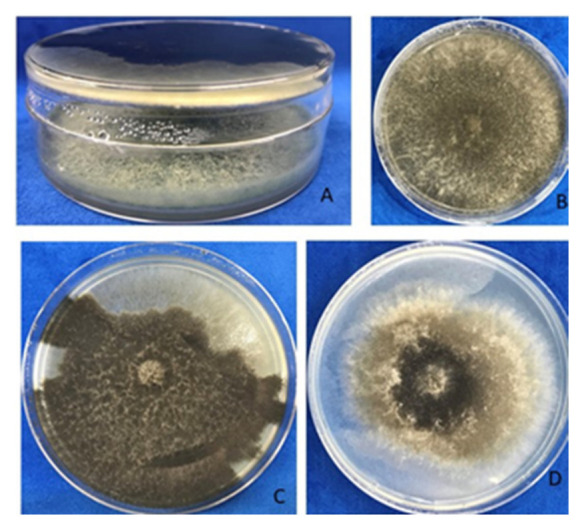
(**A**) Inverted plate set-up for VOCs-pathogen top plate and *Trichoderma* isolate at the bottom plate. (**B**) 7-day-old Control plate of *Macrophomina* (**C**) VOCs inhibition on BRIP74298_*T. peberdyi* and (**D**) VOCs inhibition on BRIP74286_*T. asperelloides*.

**Figure 6:**
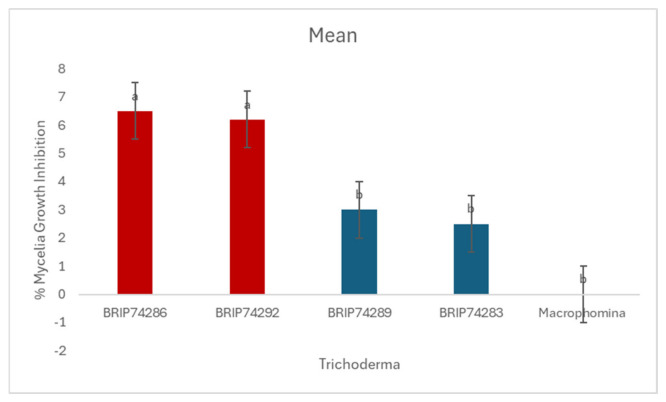
Effect of volatile organic compounds produced by *Trichoderma* isolates: BRIP74286_*T. asperelloides*; BRIP74292_*T. peberdyi*; BRIP74289_*T. asperelloides*; and BRIP74283_*T. azevedoi*, on mycelial growth inhibition of *Macrophomina*. Bars represent mean ± standard error (SE). Different letters indicate significant differences according to Tukey’s HSD test (*p* < 0.05). Red bars indicate significantly higher inhibitory isolates. The rest of the *Trichoderma* isolates did not show an inhibitory effect.

Volatile organic compounds (VOCs) produced by *Trichoderma* isolates had a highly significant effect on the mycelial growth of *Macrophomina* strain BRIP 70719 (*p* < 0.001), as shown in Figure 5 and Figure 6, indicating substantial variation in antagonistic potential among isolates.

Analysis of variance confirmed a significant effect of *Trichoderma* isolate on growth inhibition, highlighting marked differences in VOC-mediated activity across the population. Only a small subset of isolates exhibited significant inhibitory effects. In particular, *T. asperelloides* (BRIP74286) and *T. peberdyi* (BRIP74292) achieved the highest levels of inhibition, both reaching approximately 6.5%, and formed the top statistical group according to Tukey’s.

The Tukey’s HSD test was conducted, and showed that these isolates were clearly distinct from all other isolates, suggesting the production of antifungal volatile compounds and identifying them as promising candidates for VOC-based biocontrol.

A limited number of isolates displayed low but measurable inhibition, generally within the range of 1–3%. These included *T. asperelloides* (BRIP74289) with approximately 2.94% inhibition and *T. azevedoi* (BRIP74303) with approximately 2.35% inhibition, as well as BRIP74283 (*T. azevedoi*) and BRIP74298 (*T. peberdyi*). However, these isolates were not statistically different from those exhibiting no inhibition, suggesting that their VOC production is weak, inconsistent, or insufficient to suppress pathogen growth under the conditions tested.

In contrast, the majority of *Trichoderma* isolates showed no detectable inhibition via VOCs and were grouped together statistically. This indicates that VOC-mediated antagonism is not a universal trait within *Trichoderma*, but rather a highly strain-specific characteristic. Most isolates likely either do not produce antifungal volatile compounds or produce them at levels that do not result in measurable inhibition.

Overall, Tukey’s HSD analysis separated the isolates into two primary functional groups: a small group of highly active VOC producers and a large group with low or no inhibitory activity. When considered alongside the widespread occurrence of enzyme-mediated antibiosis observed in this study demonstrates that *Trichoderma* isolates employ diverse, complementary antagonistic strategies. Collectively, the findings highlight that VOC-mediated inhibition is a specialised trait restricted to a few isolates and emphasise the importance of strain-level screening for the identification of effective biological control agents.

## 4. Discussion

This study provides the first integrated, multilocus-based characterisation of indigenous *Trichoderma* populations associated with Australia’s northern cropping systems, linking phylogenetic identity with functional biocontrol traits. By combining multilocus sequencing with in vitro antagonism assays, the work moves beyond species-level reporting and demonstrates that biocontrol potential is strongly governed primarily by strain-level functional variation rather than species identity.

Multilocus analysis enabled reliable resolution of isolates into six *Trichoderma* species, with *T. azevedoi* and *T. afroharzianum* dominating across regions. While global studies often focus on a limited number of species such as *T. harzianum* and *T. virens* [39,40], these results highlight the importance of identifying locally adapted taxa suited to specific agroecosystems. In Australia, where biosecurity frameworks favour indigenous organisms [41], this strengthens the case for regionally derived biocontrol solutions. Reliable species delimitation using multilocus markers is essential in *Trichoderma* due to the limited resolution of ITS alone [18,42].

Despite a relatively low recovery frequency, the isolates obtained represent a functionally diverse subset of the rhizosphere community. Isolation bias associated with selective media and environmental variability has been shown to influence the recovery of *Trichoderma* spp. [43]. Therefore, the dataset likely reflects a functional subset rather than the full diversity present. This supports the idea that targeted isolation and screening can effectively capture biologically relevant variation without requiring exhaustive biodiversity surveys.

A key finding of this study is the high level of variability in antagonistic performance among isolates. Several isolates consistently suppressed *Macrophomina* across multiple pathogen genotypes, whereas others showed moderate or negligible effects. The absence of clear geographic clustering among top-performing isolates indicates that antagonistic capacity is not determined by origin but by intrinsic functional traits. Similar observations have been reported in other systems, where *Trichoderma* efficacy is governed by physiological and biochemical properties rather than geographic source [7,8,44]. The inclusion of multiple pathogen isolates revealed significant isolate × pathogen interactions, reinforcing the need for multi-strain screening to capture variability in pathogen response.

Functional assays further highlight the mechanistic basis of this variability. Enzyme-associated antibiosis, including protease, cellulase, and chitinase activity, was widespread and likely contributes to pathogen suppression through cell wall degradation and mycoparasitism [44]. These enzymes are well-recognised components of *Trichoderma* antagonism. However, because the assays used were semi-quantitative, these results should be interpreted as indicators of functional potential rather than precise measures of activity.

In contrast, VOC-mediated inhibition was restricted to a small subset of isolates, confirming that this mechanism is highly strain-specific. Microbial volatile compounds are recognised mediators of microbial interactions [45] and can suppress pathogens through diverse antifungal effects [46]. The variability observed here is consistent with reports that VOC production and efficacy differ substantially among *Trichoderma* strains [47]. Although the magnitude of inhibition was modest compared with direct antagonism, VOCs may still contribute to pathogen suppression in confined soil environments. Early work by Dennis and Webster [48] also demonstrated that both volatile and non-volatile metabolites contribute to *Trichoderma* antagonism.

Collectively, these findings show that *Trichoderma*-mediated biocontrol is a multifaceted and strain-dependent process, involving the combined effects of enzymatic activity, competition, and, in some cases, volatile-mediated interactions [12,44]. This complexity highlights a key limitation in approaches that rely on species identity or single-trait screening to identify effective biocontrol agents.

From an applied perspective, the results support a shift toward a structured, strain-focused selection framework that integrates phylogenetic identification with functional performance across multiple assays and pathogen genotypes. This aligns with emerging perspectives in biological control that prioritise trait-based selection and ecological fitness over taxonomic identity alone.

It is important to recognise that all experiments in this study were conducted under controlled in vitro conditions. While essential for initial screening, these assays do not capture the full complexity of field environments, including plant–microbe interactions, soil heterogeneity, and climatic variability. Therefore, the biocontrol potential observed here should be considered preliminary. Validation under greenhouse and field conditions will be required to confirm the consistency and practical relevance of selected isolates.

Overall, this study demonstrates that indigenous *Trichoderma* populations in Australia harbour significant but highly variable biocontrol potential. By shifting the focus from species-based to strain-based selection, and by integrating phylogenetic and functional data, this work provides a practical pathway for identifying locally adapted biocontrol agents and advancing sustainable management strategies for charcoal rot in northern cropping systems.

## 5. Conclusions

This study demonstrates that indigenous *Trichoderma* populations in Australia’s northern cropping region possess significant but highly variable biocontrol potential against *Macrophomina*. Multilocus analysis enabled accurate species identification, while functional screening revealed that antagonistic activity varies substantially among isolates within the same species.

The findings show that biocontrol efficacy is driven primarily by strain-level functional traits rather than species identity. Enzyme-associated antibiosis was widespread, whereas VOC-mediated inhibition was limited to a small subset of isolates, highlighting the importance of multi-trait evaluation. These results support a shift from species-based selection toward a targeted, strain-focused approach.

By integrating phylogenetic identification with functional screening, this study provides a practical framework for selecting locally adapted biocontrol candidates. This framework can guide the development of effective, region-specific biological control strategies for charcoal rot. However, validation under greenhouse and field conditions remains essential to confirm the performance and reliability of selected isolates under realistic cropping environments.

## Figures and Tables

**Table 1:** Nearest identities of *Trichoderma* isolates were determined using a pairwise similarity method based on sequences from three gene loci.

BRIP Number	Geographic Origin	Pairwise Similarity Method			Type Culture Strain Used	GenBank Accession ***			
		ITS (>76%)	*rpb2* (>99%)	*tef* (>97%)		ITS	*rpb2*	*tef-1α*	Identified *Trichoderma* Species
74282	Kingaroy Res. Station, SQ	99.5	99.2	99.6	*Trichoderma azevedoi* CEN1422 (Type)	OR787373	OR781577	OR801283	*Trichoderma azevedoi*
74283	Kingaroy Res. Station, SQ	100	99.2	99.6	*Trichoderma azevedoi* CEN1422 (Type)	OR787374	OR781578	OR801289	*Trichoderma azevedoi*
74284	Kingaroy Res. Station, SQ	100	99.2	99.6	*Trichoderma azevedoi* CEN1422 (Type)	OR787375	OR781580	OR801290	*Trichoderma azevedoi*
74285	Hermitage Res. Station, SQ	92.9	99.3	97.2	*Trichoderma neokoningii* CBS:120070 strain G.J.S. 04-216 (Type)	OR787386	OR781597	OR801300	*Trichoderma neokoningii*
74286	Bundaberg, CQ	99.8	99.9	99.5	*Trichoderma asperelloides* GJS 04-111 (Type)	OR787381	OR781601	OR801295	*Trichoderma asperelloides*
74287	Capella, CQ	100	99.2	99.6	*Trichoderma azevedoi* CEN1422 (Type)	OR787377	OR781583	OR801287	*Trichoderma azevedoi*
74288	Capella, CQ	100	100	98.9	*Trichoderma afroharzianum* GJS 04-186 (Type)	OR787361	OR781587	OR801275	*Trichoderma afroharzianum*
74289	Bundaberg, CQ	100	100	99.8	*Trichoderma asperelloides* GJS 04-111 (Type)	OR787382	OR781602	OR801296	*Trichoderma asperelloides*
74290	Brookstead, SQ	99.6	98.9	99.6	*Trichoderma azevedoi* CEN1422 (Type)	OR787369	OR781584	OR801284	*Trichoderma azevedoi*
74291	Dintonvale Road, Oakwood NNSW	100	98.4	99.8	*Trichoderma gamsii* GJS 04-09	OR787383	OR781599	OR801297	*Trichoderma gamsii*
74292	Dintonvale Road, Oakwood NNSW	99.6	99.7	**	*Trichoderma peberdyi* CEN1426 (Type)	OR787379	OR781595	OR801294	*Trichoderma peberdyi*
74293	Gwydir Highway, Long Plain NNSW	98.1	99.9	98.7	*Trichoderma afroharzianum* GJS 04-186 (Type)	OR787367	OR781588	OR801280	*Trichoderma afroharzianum*
74294	Princess Lane, Long Plain NNSW	100	98.5	98	*Trichoderma gamsii* GJS 04-09	OR787384	OR781598	OR801298	*Trichoderma gamsii*
74295	Formartin, CQ	94.6	99	97.5	*Trichoderma azevedoi* CEN1422 (Type)	OR787372	OR781585	OR801291	*Trichoderma azevedoi*
74296	Little Gully Langley, Langley Road, Balfours Peak NNSW	100	99.7	98.9	*Trichoderma afroharzianum* GJS 04-186 (Type)	OR787362	OR781593	OR801276	*Trichoderma afroharzianum*
74297	Gwydir Highway, Long Plain NNSW	99.8	99.4	98.9	*Trichoderma afroharzianum* GJS 04-186 (Type)	OR787368	OR781589	OR801279	*Trichoderma afroharzianum*
74298	Dintonvale Road, Oakwood NSW	99.6	99.6	96.7	*Trichoderma peberdyi* CEN1426 (Type)	OR787380	OR781596	OR801293	*Trichoderma peberdyi*
74299	Moree, NSW	99.1	99.2	99.6	*Trichoderma azevedoi* CEN1422 (Type)	OR787370	OR781581	OR801285	*Trichoderma azevedoi*
74300	Coomooma, Moree, NSW	99.8	99.6	98.5	*Trichoderma afroharzianum* GJS 04-186 (Type)	OR787366	OR781594	OR801277	*Trichoderma afroharzianum*
74301	Moree, NSW	100	99.3	98.8	*Trichoderma afroharzianum* GJS 04-186 (Type)	OR787363	OR781591	OR801281	*Trichoderma afroharzianum*
74302	Capella, CQ	100	99.2	99.6	*Trichoderma azevedoi* CEN1422 (Type)	OR787376	OR781582	OR801288	*Trichoderma azevedoi*
74303	Capella, CQ	95.5	99.3	99.6	*Trichoderma azevedoi* CEN1422 (Type)	OR787378	OR781579	OR801286	*Trichoderma azevedoi*
74304	Capella, CQ	100	99.9	98.9	*Trichoderma afroharzianum* GJS 04-186 (Type)	OR787364	OR781590	OR801278	*Trichoderma afroharzianum*
74305	Clermont, CQ	100	100	98.5	*Trichoderma afroharzianum* GJS 04-186 (Type)	OR787365	OR781592	OR801282	*Trichoderma afroharzianum*
74306	Clermont, CQ	98.9	99	97.5	*Trichoderma azevedoi* CEN1422 (Type)	OR787371	OR781586	OR801292	*Trichoderma azevedoi*
DA1316 *	Dintonvale Road, Oakwood NNSW	98.7	98.5	100	*Trichoderma gamsii* GJS 04-09	OR787385	OR781600	OR801299	*Trichoderma gamsii*

* Material stored at UniSQ and was not deposited to BRIP due to culturing challenges. ** PCR and sequencing not completed in time due to time restrictions. *** Sequences submitted to NCBI Genbank.

**Table 2:** Qualitative and quantitative agar assays for biocontrol mechanisms of *Trichoderma* isolates for the detection of (a) protease, (b) endoglucanase, (c) chitinase, (d and e) phosphate solubilization, (f) lignin and manganese peroxidase, (g) laccase and volatile organic compounds (VOCs), (h) inhibitory VOCs.

Trichoderma Isolates	Skim Milk Agar (a)	CMC Agar (b)	Chitin Agar (c)	Pikovskaya’s Medium (d)	NBRIP Agar (e)	Bromophenol Blue Plate (f)	Azure B Agar (g)	Inhibitory VOCs (h)
	Antibiosis (Lytic Enzymes)	Antibiosis (Lytic Enzymes)	Antibiosis (Lytic Enzymes)	Induced Plant Resistance (Biofertilization)	Induced Plant Resistance (Biofertilization)	Antibiosis (Lytic Enzymes)	Antibiosis (Lytic Enzymes)	Antibiosis (Inhibitory Volatile Compounds)
*T. azevedoi* (BRIP74282)	+	+	+	−	−	−	−	−
*T. acevedoi* (BRIP 74283)	+	+	+	−	−	−	−	−
*T. acevedoi* (BRIP 74284)	+	+	+	−	−	−	−	−
*T. neokoningii* (BRIP 74285)	+	+	+	−	−	−	−	−
*T. asperelloides* (BRIP 74286)	+	+	−	−	−	−	−	+
*T. azevedoi* (BRIP 74287)	+	+	+	−	−	−	−	−
*T. afroharzianum* (BRIP 74288)	+	−	−	−	−	−	−	−
*T. asperelloides* (BRIP 74289)	+	+	−	−	−	−	−	+
*T. azevedoi* (BRIP 74290)	−	+	−	−	−	−	−	−
*T. gamsii* (BRIP 74291)	+	+	+	−	−	−	−	−
*T. peberdyi* (BRIP 74292)	+	−	+	−	−	−	−	+
*T. afroharzianum* (BRIP 74293)	+	+	+	−	−	−	−	−
*T. gamsii* (BRIP 74294)	−	−	−	−	−	−	−	−
*T. azevedoi* (BRIP 74295)	+	+	−	−	−	−	−	−
*T. afroharzianum* (BRIP 74296)	+	+	+	−	−	−	−	−
*T. gamsii* (DA 1316)	+	+	+	−	−	−	−	−
*T. afroharzianum* (BRIP 74297)	+	+	+	−	−	−	−	−
*T. peberdyi* (BRIP 74298)	+	−	+	−	−	−	−	−
*T. azevedoi* (BRIP 74299)	+	+	+	−	−	−	−	−
*T. afroharzianum* (BRIP 74300)	+	+	+	−	−	−	−	−
*T. afroharzianum* (BRIP 74301)	+	+	+	−	−	−	−	−
*T. azevedoi* (BRIP 74302)	+	+	+	−	−	−	−	−
*T. azevedoi* (BRIP 74303)	+	+	+	−	−	−	−	+
*T. afroharzianum* (BRIP 74304)	+	+	+	−	−	−	−	−
*T. afroharzianum* (BRIP 74305)	+	+	+	−	−	−	−	−
*T. azevedoi* (BRIP 74306)	+	−	+	−	−	−	−	−

## Data Availability

The data supporting the findings of this study are stored in the University of Southern Queensland’s designated data repositories and are available upon reasonable request.

## Supplementary Materials

The following supporting information can be downloaded at https://www.mdpi.com/article/10.3390/cimb48070654/s1.

## Abbreviations

The following abbreviations are used in this manuscript:

ANOVA	Analysis of Variance
BI	Bayesian inference
bp	Base pairs
BRIP	Plant Pathology Herbarium isolate code (Queensland)
CMC	Carboxymethyl cellulose
CQ	Central Queensland
CRD	Completely Randomised Design
DNA	Deoxyribonucleic acid
ddH_2_O	Double distilled water
GRDC	Grains Research and Development Corporation
ICTT	International Commission on *Trichoderma* Taxonomy
ITS	Internal transcribed spacer
MCMC	Markov Chain Monte Carlo
ML	Maximum likelihood
NBRIP	National Botanical Research Institute’s phosphate medium
NCBI	National Center for Biotechnology Information
NNSW	Northern New South Wales
PDA	Potato dextrose agar
PDB	Potato dextrose broth
PCR	Polymerase chain reaction
PGI	Percentage growth inhibition
PI	Percent inhibition
Qld	Queensland
SQ	Southern Queensland
VOCs	Volatile organic compounds
WA	Water agar
