# Supplementary material for: Multilocus Identification of Indigenous Trichoderma Isolates and Their Biocontrol Mechanisms Against Macrophomina in Northern Australia
Source: Curr Issues Mol Biol. 2026 Jun 25;48(7):654. doi: 10.3390/cimb48070654 (PMC13406979; doi:10.3390/cimb48070654)
Supplement: Supplementary file 1 [file cimb-48-00654-s001.zip › cimb-4347302-supplementary.pdf]

Supplementary Table S1. GPS coordinates of sampling sites in the Northern Cropping Region for *Trichoderma* isolation.

Address	Region	Latitude	Longitude
Dintonvale Road, Oakwood NSW	NNSW	-29.602314	151.115845
Little Gully Langley, Langley Road, Balfours Peak NSW	NNSW	-29.444444	150.817778
Little Gully Langley, Langley Road, Balfours Peak NSW	NNSW	-29.444722	150.815556
Litte Gully Langley, Langley Road, Balfours Peak NSW	NNSW	-29.444722	150.815556
Litte Gully Langley, Langley Road, Balfours Peak NSW	NNSW	-29.442222	150.815833
The Glen Penberthy, Gwydir Highway, Long Plain NSW	NNSW	-29.765556	151.246389
The Glen Penberthy, Princes Lane, Long Plain NSW	NNSW	-29.771944	151.261667
The Glen Penberthy, Gwydir Highway, Long Plain NSW	NNSW	-29.770000	151.253333
Budore, Delungra NSW	NNSW	-29.665000	150.887778
Budore, Delungra NSW	NNSW	-29.665000	150.885000
Budore, Delungra NSW	NNSW	-29.661667	150.879722
Gwydir Highway, Long Plain NSW	NNSW	-29.765556	151.246389
Princes Lane, Long Plain NSW	NNSW	-29.771944	151.261667
Gwydir Highway, Long Plain NSW	NNSW	-29.771000	151.253334
Litte Gully Langley, Langley Road, Balfours Peak NSW	NNSW	-29.444444	150.817778
Dintonvale Road, Oakwood NSW	NNSW	-29.617500	151.076944
Dintonvale Road, Oakwood NSW	NNSW	-29.660833	151.078889
Dintonvale Road, Oakwood NSW	NNSW	-29.615278	151.075833
Moree, NSW	NNSW	-29.461720	149.840715
Coomooma, Moree, NSW	NNSW	-29.400101	149.513723
Long Plain Ln, Inverell NSW	NNSW	-29.760278	151.230000
Killarney East, Gydir, Long Plain NSW	NNSW	-29.760556	151.253889

Mullaley, NSW	NNSW	-31.097655	149.911522
Bourbah, Mullaley NSW	NNSW	-31.236667	149.917222
Tambar Springs, NSW	NNSW	-31.242222	149.956111
Garah, NSW	NNSW	-28.875950	149.595130
Liverpool Plains, NSW	NNSW	-31.470190	150.394276
Ellangowan, QLD	SQ	-27.931900	151.684400
Ellangowan, QLD	SQ	-27.933500	151.684500
Yangan, QLD	SQ	-28.193453	152.129527
Kingaroy, QLD	SQ	-26.559516	151.839075
Dalby, QLD	SQ	-27.025660	151.097460
Brookstead,Qld	SQ	-27.757619	151.469819
Gore Hwy, Brookstead Qld	SQ	-27.757851	151.485731
Warra, QLD	SQ	-26.928875	150.919171
Toowoomba Cecil Plains, QLD	SQ	-27.542582	151.517204
Toowoomba Cecil Plains, QLD	SQ	-27.528198	151.584821
Bongeen, QLD	SQ	-27.522359	151.445858
Bowenville, QLD	SQ	-27.505144	151.391710
Formartin, QLD	SQ	-27.467625	151.426514
Bundaberg, QLD	CQ	-24.865325	152.351679
Capella, QLD	CQ	-28.552126	150.290503
Capella, QLD	CQ	-28.552126	150.290503
Capella, QLD	CQ	-22.933737	148.114927
Capella, QLD	CQ	-22.933681	148.002995
Capella, QLD	CQ	-28.552126	150.290503
Clermont, QLD	CQ	-22.543156	147.663093
Clermont, QLD	CQ	-22.471918	147.700653
Carina Downs, Springsure QLD	CQ	-24.240556	148.477778
Boongulla, Springusre QLD	CQ	-23.868417	148.413772
Long Mill, Gindie QLD	CQ	-23.741389	148.333333
Amah Rd, Capella QLD	CQ	-23.246944	148.068581
Lawrence Lane Rd, Capella QLD	CQ	-23.138333	148.061667
Bungarra, Clermont QLD	CQ	-22.669786	147.791417
Glenmore Downs, Clermont QLD	CQ	-22.588250	147.638378

Supplementary Table S2. Primer sequences and PCR amplification conditions used in the study.

Locus Name	Primer Name	Sequences	Amplification Conditions
Internal Transcribed Spacers (ITS)	ITS5	5′ GGAAGTAAAAGTCGTAACAAGG 3′	initial denaturation at 94°C for 3min, followed by 34 cycles of denaturation at 95°C for 1min, annealing at 56°C for 45s, and extension at 72°C for 90s, and then a final extension at 72°C for 10min
	ITS4	5′ TCCTCCGCTTAT TGATATGC 3′	
RNA Polymerase II (*rpb2*) gene	fRPB2-5	5′-GAYGAYMGWGATCAYTTYGG-3′	start step of 5 min at 94°C, followed by 5 cycles of 45s at 94°C, 45 s at 60°C annealing temperature, and 2 min at 72°C; 5 cycles of 45 s at 94°C, 45s at 58 °C annealing temperature, and 2 min at 72°C; 30 cycles of 45s at 94°C, 45 s at 54°C annealing temperature, and 2 min at 72°C followed by a final step of 8min at 72°C
	fRPB2-7cr	5′-CCCATRGCTTGTYYRCCCAT-3′	
Translation Elongation Factor (*tef - 1α*) gene	ef1	5′-CATCGAGAAGTTCGAGAAGG-3′	initial denaturation at 94°C for 2min, followed by 35 cycles of denaturation at 95°C for 1min, annealing at 55°C for 30s, and extension at 72°C for 1min, and then a final extension at 72°C for 10min
	ef2	5′-TACTTGAAGGAACCCTTA-3′	

Supplementary Table S3. Details of bioassays used in this study.

Assay	Method	Result Interpretation
Skim Milk Agar Assay	Ten microlitres of each *Trichoderma* isolate suspended in sterile distilled water was spot-plated on 1.5% skimmed milk agar and 1.5% agar, and incubated at 25°C for 98h.	The formation of clear zones in the agar indicates protease activity
Carboxymethyl Cellulose Agar Assay	Ten microlitres of each *Trichoderma* isolate suspended in sterile distilled water were spot-plated on CMC agar, and the plates were incubated at 25°C for	CMC degradation positive appears as a yellow opaque area against a red colour for undegraded CMC. The assay aimed at identifying

	98 hours. The plates were flooded with 0.1% Congo red for 15 to 20 minutes and then with 1M NaCl for 15 to 20 minutes.	cellulolytic microorganisms, in this case *Trichoderma* isolates, that play an important role in recycling cellulose in the biosphere, by catalysing the hydrolysis of cellulose into sugars, the most abundant carbohydrate produced by plants
Chitin Agar Assay	*Trichoderma* plugs of 5mm dia were inoculated onto the chitin agar medium and incubated at 25±2°C for 3-5 days, and were observed for purple coloured zone formation	Chitinase activity was determined by the diameter of the purple coloured zone after 3 days of incubation in the region of chitin utilisation on the colloidal chitin-supplemented agar medium.
Phosphate Solubilizing Agar Assay	Fresh 5mm plug of each *Trichoderma* species was screened for its phosphate-solubilising activity in vitro using Pikovskaya's medium or NBRIP medium due to the production of organic acids in to the surrounding medium. Plates were incubated at 25°C for 4-7 days.	The halo/clear zones around mycelial growth on the plates served as an indicator of a potential phosphate solubiliser.
Bromophenol Blue Plate Agar Assay	Plates were inoculated with 5mm plugs of Trichoderma isolates and incubated at 25°C for 4-7 days.	The degree of decolourisation of the dye was visually examined by noticing clear halo zones around laccase-producing isolates.
Azure B Agar Assay	Plugs (5mm) of *Trichoderma* isolates were aseptically transferred to the agar plates containing 0.01% yeast, 0.01% peptone, 0.01 % Azure B and 1% glucose, and incubated at 25°C in dark conditions for 4-7 days.	Clearance of blue colour in the assay plates confirmed the production of lignin and manganese peroxidase.
Growth inhibition by Volatile Organic Compounds (VOCs)	The inverted plate technique was used to assess volatile compounds produced by *Trichoderma.* Agar plugs (2 mm) of each *Trichoderma* isolate and *Macrophomina* (BRIP70719) were placed at the centre of separate PDA plates. Plates containing *Macrophomina* were inverted over Trichoderma plates and sealed with two layers of parafilm to prevent compound loss, with *Trichoderma* on the lower plate	The colony diameter of the pathogen was measured after 7 days and compared to the control. The percentage inhibition of mycelial growth (PGI) of *Macrophomina* due to the volatile compounds was calculated using the following formula: $PGI = [(C - T) / C] \times 100$, where PGI represents the growth inhibition of the pathogen (%), C is the radial growth of the control

	and *Macrophomina* on the upper plate.<br>Control plates consisted of *Macrophomina* inverted over PDA-only plates and incubated at 25 °C. Each treatment was replicated five times.	*Macrophomina*, and T is the radial growth of the treated *Macrophomina*.
